# Enhanced Multiscale Principal Component Analysis for Improved Sensor Fault Detection and Isolation

**DOI:** 10.3390/s22155564

**Published:** 2022-07-26

**Authors:** Byanne Malluhi, Hazem Nounou, Mohamed Nounou

**Affiliations:** 1 Chemical Engineering Program, Texas A&M University at Qatar, Doha P.O. Box 23874, Qatar; byanne.malluhi@qatar.tamu.edu; 2 Electrical and Computer Engineering Program, Texas A&M University at Qatar, Doha P.O. Box 23874, Qatar; hazem.nounou@qatar.tamu.edu

**Keywords:** multiscale PCA, process monitoring, fault detection, fault isolation, sensor faults, wavelet analysis

## Abstract

Multiscale PCA (MSPCA) is a well-established fault-detection and isolation (FDI) technique. It utilizes wavelet analysis and PCA to extract important features from process data. This study demonstrates limitations in the conventional MSPCA fault detection algorithm, thereby proposing an enhanced MSPCA (EMSPCA) FDI algorithm that uses a new wavelet thresholding criterion. As such, it improves the projection of faults in the residual space and the threshold estimation of the fault detection statistic. When tested with a synthetic model, EMSPCA resulted in a 30% improvement in detection rate with equal false alarm rates. The EMSPCA algorithm also relies on the novel application of reconstruction-based fault isolation at multiple scales. The proposed algorithm reduces fault smearing and consequently improves fault isolation performance. The paper will further investigate the use of soft vs. hard wavelet thresholding, decimated vs. undecimated wavelet transforms, the choice of wavelet decomposition depth, and their implications on FDI performance.The FDI performance of the developed EMSPCA method was illustrated for sensor faults. This undertaking considered synthetic data, the simulated data of a continuously stirred reactor (CSTR), and experimental data from a packed-bed pilot plant. The results of these examples show the advantages of EMSPCA over existing techniques.

## 1. Introduction

In pursuit of a futuristic industry 4.0, data-driven techniques are receiving more and more attention. Using process data and efficient data analytic algorithms, monitoring technologies can more effectively identify faults for more complex processes. This is especially important for the safe, efficient, and reliable operation of industrial processes that rely on sensor data in their monitoring and control systems. Fault detection and isolation methods are generally categorized as model-based, data-based, or a hybrid of the two [1]. Principle component analysis (PCA) is among the most prominent data-based techniques. Multiscale PCA (MSPCA) is a well-established extension of PCA, and it is progressively being used in the process monitoring literature [2,3,4,5].

Multiscale PCA couples the monitoring capability of PCA with the advantages of wavelet-based multiscale representation of data: A PCA model reduces the dimensionality of the data by exploiting the correlation among the different variables; it captures the main variation in the data with transformed variables, called “principle components”. Wavelet analysis decomposes a signal into “detail” and “approximate” coefficients, which capture the high and low frequencies of a signal. MS analysis can separate stochastic and deterministic features in process data and approximately de-correlate auto-correlated signals [6,7]. MSPCA is among the many extensions of PCA. Other extensions include dynamic PCA [8], probabilistic PCA [9], kernel PCA with kernel density estimation [10], and Bayesian network PCA [11]. Such extensions address issues arising from PCA’s underlying assumptions of linear, stationary, and multivariate normal data with Gaussian noise.

Wavelet coefficient selection, also called wavelet-based signal denoising, is paramount to the accuracy of MS-based fault detection algorithms. MSPCA uses PCA to select coefficients at every scale that violate the threshold limits obtained from the Q/T2 statistics [7]. One objective of this work is to improve fault detection of MSPCA by enhancing the coefficient selection steps in the training and testing phases of the algorithm. Authors in [12] highlight the issue of poor detection performance when using the conventional coefficient selection criteria. In their work, they recommend a combination of statistics to improve the accuracy of wavelet coefficient selection. Other works have proposed different methods for coefficient selection. For example, authors in [13] use Shannon and Tsillis entropies as thresholders to select coefficients that maximize the signal to noise ratio (SNR). Authors in [14] employ a multiscale data reconciliation technique through Q-R factorization at multiple scales. The works of [15,16,17] use empirical mode decomposition and empirical wavelet transform, which decompose signals according to the frequency content of the data.

Furthermore, other implementations of the MSPCA algorithm in the literature have modified how different scales are processed by the wavelet coefficient selection steps. For example, the authors in [18] only model the scales that contain underlying process changes. They selectively remove scales that contain little or no information by a quantitative analysis of their energies. In the work of [5], all the coefficients in the final approximate scale are selected, and thus, the approximate scale is not subjected to any PCA modeling. However, in the conventional algorithm, all the scales are modeled irrespective of the information they contain. Despite these modifications from the conventional approach, no work directly compares their implications on fault detection accuracy. This research aims to fill this gap and demonstrate the limitations of the current MSPCA coefficient selection criteria by analyzing its impact on the fault residual projection and threshold estimation. This will motivate the enhanced coefficient selection criteria, which combines ideas from [5,18] in the way detail and approximate scales are processed within the coefficient selection steps.

A second objective of this work is to maximize the advantages of MSPCA to improve the accuracy of reconstruction-based PCA fault isolation. Reconstruction-based isolation approaches involve PCA reconstruction, which use the PCA model to compute a variable from the other remaining variables. Previous literature demonstrates the implementation of contribution plot PCA isolation at multiple scales, but to the best of the authors’ knowledge, no work has extended it to reconstruction-based PCA isolation despite its superior performance with respect to smearing [4,19,20]. Although the proposed isolation approach can also handle process faults through its blueprint representations at the different scales, this work will focus on identifying sensor faults in batch processes. The algorithm can be performed in real time for online monitoring by applying a moving window, as in the works of [21,22], or by applying just-in-time feature analysis, as in the works of [23,24]. Furthermore, the algorithm can be extended to fault diagnosis by the recursive implementation of PCA on the data, as in the works of [25], which help obtain characteristic diagnostic features.

A third objective of this work is to illustrate the practical advantages of using the undecimated or stationary wavelet transform over the commonly used decimated wavelet transform in fault detection. The decimated wavelet transform reduces the number of samples by half at each subsequent coarser scale to remove data redundancy [26]. The undecimated wavelet transform retains the same number of samples at each subsequent coarser scale [26]. The DWT will demonstrate the extent at which the advantages of data redundancy can compromise FDI accuracy. This study will use the average computational time to quantitatively analyze the trade-off between accuracy and computational effort.

This paper is organized as follows: Section 2 will provide background on PCA fault detection and isolation, multiscale analysis, and the conventional MSPCA algorithm. Section 3 will describe the new coefficient selection criterion and the proposed EMSPCA algorithm. Section 4 will compare the coefficient selection approaches of MSPCA and EMSPCA for fault detection. Section 5 will assess the EMSPCA isolation performance. Section 6 will illustrate the impact of the decimated and undecimated wavelet transforms on the FDI performance. Section 7 will compare the computational time and detection accuracy of the algorithms. Section 7 and Section 8 will assess the EMSPCA algorithm using simulated CSTR reactor data and experimental data from a packed-bed pilot plant. Finally, Section 9 will conclude the findings of this work and suggest future directions.

## 2. Theory and Background

### 2.1. PCA-Based Fault Detection and Detectability

PCA decomposes a sample x ($x\in R^{mx1}$) into a modeled ($\hat{x}$) and un-modeled component ($\tilde{x}$) [27],

(1) $$x=Cx+\left(I-C\right)x=\hat{x}+\tilde{x},$$

where C is the transformation matrix that projects the data onto the principal component (PC) subspace. I−C, also denoted by $\tilde{C}$, is the orthogonal transformation which projects the data onto the un-modeled subspace or the residual subspace [27]. The matrix ***C*** is computed by

(2) $$C=\hat{P}{\hat{P}}^{T},$$

where ($\hat{P}\in R^{mxl}$) is a subset of the full-rank eigenvector matrix (P) that is computed by the eigen-decomposition of the training data covariance matrix [27],

(3) $$R\equiv {\mathrm{XX}}^{T}/n-1=P\Lambda P^{T}.$$

In Equation (3), X is the training data with *m* variables and *n* samples. Each variable is normalized to zero mean and unit variance. The diagonal matrix Λ contains the eigenvalues, and the matrix P contains the eigenvectors in columns. The matrix $\hat{P}$ contains *l* loading vectors that capture most of the variations in the data. Different methods for computing *l* exist, such as the Scree test [28], cross validation [29,30], and cumulative percent variance (CPV) [21].

Common PCA detection statistics include Q, $T^{2}$, and φ. The Q statistic is the sum of squared errors and it is highly sensitive to changes in the correlation structure of the data. The Hoteling’s $T^{2}$ statistic measures the abnormal variations within the principal components or scores, while the φ statistic is a combination of the $T^{2}$ and Q statistics, accounting for variability in the entire measurement space (the residual and PC space) [31]. Each detection statistics can be computed by the general formula, $\mathrm{statistic}\left(x\right)=x^{T}\mathrm{Mx}$, where M equals $\tilde{C}$, or $\hat{P}{\hat{\Lambda }}^{-1}{\hat{P}}^{T}$ or $\left(\hat{P}{\hat{\Lambda }}^{-1}{\hat{P}}^{T}\right)/\tau^{2}+\tilde{C}/\delta^{2}$ for the Q, $T^{2}$, and φ statistic, respectively, where $\delta^{2}$ and $\tau^{2}$ are the statistical thresholds for the Q and $T^{2}$ indices [31]. This paper will conduct its analysis based on the Q statistic because of the desired sensitivity towards the correlation structure of the data which covers a wide range of abnormal conditions, including sensor faults.

Fault detection performance relies on satisfying the conditions for detectability [27]. Consider a data sample x, divided into non-faulty and faulty components as follows: $x=x^{*}+f\xi$, where *f* and $\xi_{i}$ represent the faulty component’s magnitude and direction, respectively. Also, consider their projection onto the residual subspace, as follows:

(4) $$\tilde{x}={\tilde{x}}^{*}+\tilde{f}{\tilde{\xi }}_{i}.$$

To guarantee fault detection by the Q statistic, it is necessary that ${\tilde{\xi }}_{i}\neq 0$ and $|\tilde{f}|>2\delta$ (where δ is the threshold limit) [27]. The magnitude of the fault projection $|\tilde{f}|$ is more important than the actual fault size |f| for PCA Q-statistic fault detection. Therefore, to improve fault detection performance in MSPCA, the fault projection in the residual space $|\tilde{f}|$ must be preserved by the reconstructed PCA model. The noise levels must also be low enough to avoid false alarms, which will enable capturing smaller faults with lower thresholds (δ). This work computes both $|\tilde{f}|$ and δ to examine detection accuracy.

### 2.2. PCA-Based Fault Isolation

This work will employ the reconstruction-based isolation technique and compare it with the complete decomposition technique (also called contribution plots). Each approach belongs to a category of PCA isolation methods described in this section.

#### 2.2.1. General Decomposition Methods

General decomposition methods involve splitting a detection statistic into different variable contributions towards a fault. The contribution of a variable *i* towards a faulty sample x is computed by the following [32],

(5) $$GD_{i}^{index}=x^{T}M^{1-\beta }\xi_{i}\xi_{i}^{T}M^{\beta }x,$$

where β is an arbitrary parameter between 0 and 1, ${\tilde{\xi }}_{i}$ is a direction vector representing the ith column of the m×m identity matrix, and M is a formula determined by the detection statistic used. $M=\tilde{C}$ for the Q statistic, $M=\hat{P}{\hat{\Lambda }}^{-1}{\hat{P}}^{T}$ for the T2 statistic, and $M=\left(\hat{P}{\hat{\Lambda }}^{-1}{\hat{P}}^{T}\right)/\tau^{2}+\tilde{C}/\delta^{2}$ for the φ statistic [32]. The partial decomposition (PD) contribution is obtained when β=0 or when β=1. The PD index was developed for the $T^{2}$ statistic by Nomikos [33]. However, it is not preferred for isolation because of the counter-intuitive negative contribution values and the asymmetry in its form that does not guarantee a positive semi-definite matrix [32].

The Complete Decomposition (CD) contribution, popularly known as contribution plots, is obtained when β=1/2. The complete decomposition (CD) contribution of variable *i* at a particular sample x has the following equation [32],

(6) $$CD_{i}^{index}=x^{T}M^{1/2}\xi_{i}\xi_{i}^{T}M^{1/2}x={\left(\xi_{i}^{T}M^{1/2}x\right)}^{2}.$$

The CD index decomposes a particular statistic into its contributing components, such that the sum of all variable contributions yields the value of the detection statistic itself. The application of contribution plots for statistical process control (SPC) was introduced for batch processes by the authors in [33,34]. It has since been successfully implemented in many industrial applications. The authors in [35] used Q contributions to identify faulty sensors in air handling units, and authors in [36] use both $T^{2}$ and Q contributions to identify faults that occur in the rolling production of seamless tube process [36]. Due to its wide use and popularity, this work will consider the CD contributions or contribution plots as a benchmark for comparison.

#### 2.2.2. Reconstruction Methods

The reconstruction method is a category which involves PCA reconstruction (not to be confused with wavelet reconstruction). PCA reconstruction is the estimation of a variable using the PCA model and the other remaining variables (omitting the variable being estimated) under the objective of minimizing the error [27]. Reconstruction-based (RB), angle-based contribution (ABC), and fault identification index (FII) are all “reconstruction” type isolation indices [32].

When measurements of the faulty variable are correctly reconstructed, the faulty sample x becomes fault-free, as illustrated by,

(7) $$x^{ri}=x-f\xi_{i}$$

where $x^{ri}$ represents the sample with reconstructed variable *i*, x is the faulty sample, and $f{\tilde{\xi }}_{i}$ is the faulty component [27]. This interpretation works well in the case of univariate sensor faults. Each variable reconstruction will result in new estimates of the $T^{2}$, Q, or φ statistic [27]. The variable reconstruction that significantly lowers the detection statistic value from its value before reconstruction will have a higher fault isolation index. The detection statistic for the reconstructed sample ($x^{ri}$) is ${∥M^{1/2}x^{ri}∥}^{2}$, which can be expressed as [32]

(8) $${∥M^{1/2}x^{ri}∥}^{2}={∥M^{1/2}x∥}^{2}-{∥M^{1/2}f\xi_{i}∥}^{2},$$

where ${∥M^{1/2}x∥}^{2}$ is the detection statistic of the original faulty sample, and ${∥M^{1/2}f\xi_{i}∥}^{2}$ is the faulty contribution towards the statistic. The latter term represents the reconstructed-based (RB) contribution for a variable *i*. The RB contribution was established in 2009 for all detection statistics [20]. The angle-based contribution (ABC) and the sensor validity index (SVI) are obtained by rearranging the above equation and dividing by the detection statistic of the testing sample [19].

This work will use the RB contribution because of the statistical simplicity that comes with its definition as a difference between detection indices (rather than the ratios). The contribution plot isolation will be utilized as a benchmark for comparison. Thus, the RB and CD contributions, written in terms of the sample vector, x, the residual model, $\tilde{C}$, and the fault direction, ${\tilde{\xi }}_{i}^{T}$, are as follows [20]:

(9) $$RB_{i}^{Q}={\left(\xi_{i}^{T}\tilde{C}x\right)}^{2}/{\tilde{c}}_{ii}\;\mathrm{and}\;CD_{i}^{Q}={\left(\xi_{i}^{T}\tilde{C}x\right)}^{2}.$$

The faulty variable is determined from the relative magnitudes of all variable fault isolation contributions. The faulty variable is the largest contributor towards the isolation index [4,37,38,39].

#### 2.2.3. Smearing Effect

All PCA fault isolation indices suffer from a phenomenon called smearing. Smearing occurs when a fault in variable *j* impacts the fault isolation indices of the other variables i≠j. When the impact is sufficiently large, the contribution of a non-faulty variable *i* is greater than the contribution of the faulty variable *j*, leading to incorrect isolation [40]. For example, the authors in [19] demonstrate how the smearing effect led to a misdiagnosis in a CSTR reactor application.

Smearing is caused by the very nature of PCA which relies on the interdependencies between variables that project data onto dimensions of lower rank. The work of [20] examines smearing in both the contribution plot and reconstruction-based (RB) isolation methods. They demonstrate that RB can guarantee fault diagnosis for large enough faults (despite smearing effects) while the traditional contribution plot cannot. This suggests that the RB isolation approach is more resistant to smearing than the CD approach, which motivates our implementation of the multiscale fault isolation algorithm with the RB approach.

### 2.3. Wavlet-Based Analysis of Data

Wavelet-based multiscale analysis uses wavelet and scaling functions to represent a signal at multiple scales. Well-known examples of wavelet functions include the Haar, Daubechies, Coiflet, and Symlet functions [41]. In this work, the Haar wavelet function is used for its mathematical simplicity. A mother wavelet function can be expressed as [7],

(10) $$\psi_{sk}\left(t\right)=1/\sqrt{s}\psi \left(t/s-k\right),$$

where, *s* and *k* are dilation and translation parameters. For practical purposes, the wavelet and scaling functions are discretized dyadically by defining the dilation parameter as $s=2^{j}$ [7]. Consequently, the wavelet function and the orthornormal scaling function are expressed as

(11) $$\psi_{jk}\left(t\right)=\sqrt{2^{-j}}\psi \left(2^{-j}t-k\right),\;\varphi_{jk}\left(t\right)=\sqrt{2^{-j}}\varphi \left(2^{-j}t-k\right),$$

**Figure 1:**
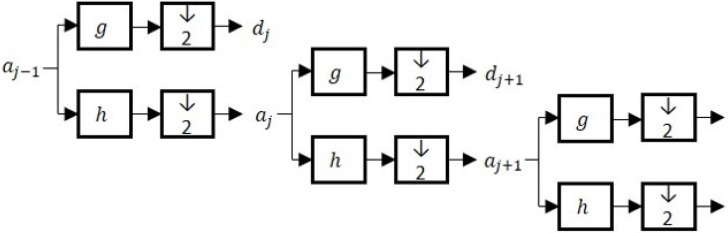
Schematic diagram of the decimated wavelet transforms (DWT), where *h* and *g* represent the high- and low-pass filters, and *a* and *d* represent the approximate and detail signals.

A filter bank structure with low-pass and high-pass filters derived from the scaling and wavelet functions, respectively, are used to implement the decimated wavelet transform (DWT) [41,42]. The high-pass filter has an impulse response *g* derived from the wavelet functions ψ(t), and the low-pass filter has an impulse response *h* derived from the scaling function φ [43]. The DWT algorithm relies on repeatedly applying the filters *h* and *g* and the down-samplers (↓2) at each scale as illustrated in Figure 1.

**Figure 2:**
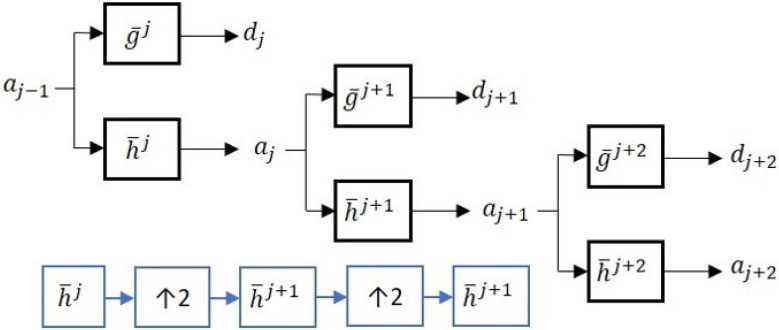
Schematic diagram of the undecimated wavelet transform (UWT); *h* and *g* are high- and low-pass filters; *a* and *d* represent the approximate and detail signals.

As a result of down-sampling in the DWT, the number of samples is halved at every ensuing scale, and the location of a feature impacts its representation at multiple scales [44]. These issues do not arise in the undecimated (or stationary) wavelet transform (UWT), because the same number of samples is maintained at every scale. This could also be an advantage for data-driven techniques that require large data sets for statistical inference. UWT can be implemented by applying low-pass and high-pass filters as shown in Figure 2.

**Figure 3:**
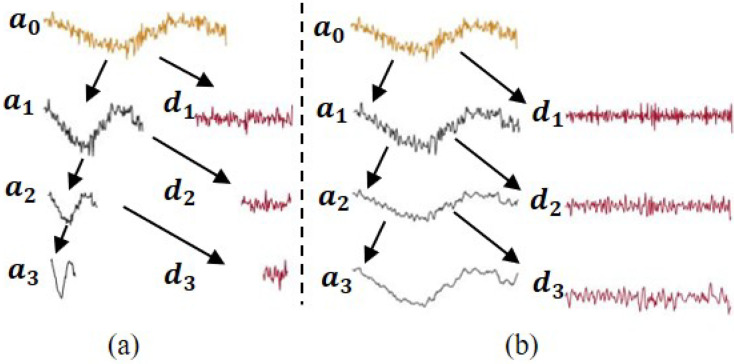
(**a**) Discrete/DWT decomposition and (**b**) stationary/UWT decomposition.

As Figure 2 indicates, the UWT up-samples the low-pass and high-pass filters at every subsequent coarser scale. Figure 3 compares the representation of a noisey sine wave signal using DWT and UWT.

To reconstruct the decomposed signals back to the time domain, all detail signals and the final approximate signal are added. The decimated and the undecimated wavelet decompositions set up the framework for MSPCA monitoring, briefly described in the next section.

### 2.4. MSPCA Algorithm

The MSPCA algorithm consists of two phases, training and testing. The training algorithm computes PCA models and detection thresholds for all detail scales, and the final approximate scale of the decomposed fault-free data [7]. In the training phase, when one threshold violation occurs, the algorithm will select all coefficients of that scale for wavelet reconstruction. After wavelet reconstruction, the algorithm computes the PCA model and detection threshold for the final reconstructed signals.

**Figure 4:**
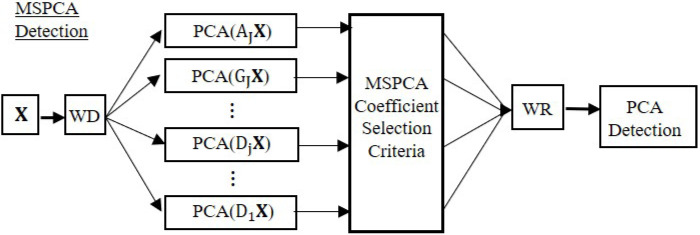
MSPCA fault detection algorithm.

The MSPCA testing algorithm uses the PCA models to obtain the residuals at each scale of the decomposed testing data. Then, the detection thresholds are used to identify the “significant” coefficients for reconstruction. A schematic of the MSPCA detection algorithm is demonstrated in Figure 4.

The MSPCA coefficient selection criteria determines which training and testing samples are retained for wavelet reconstruction (WR) [7]. A different coefficient selection scheme, which brought forth significant improvements, motivates the enhanced multiscale PCA (EMSPCA) algorithm.

## 3. New Coefficient Selection Criterion and Enhanced MSPCA (EMSPCA) Algorithm

**Figure 5:**
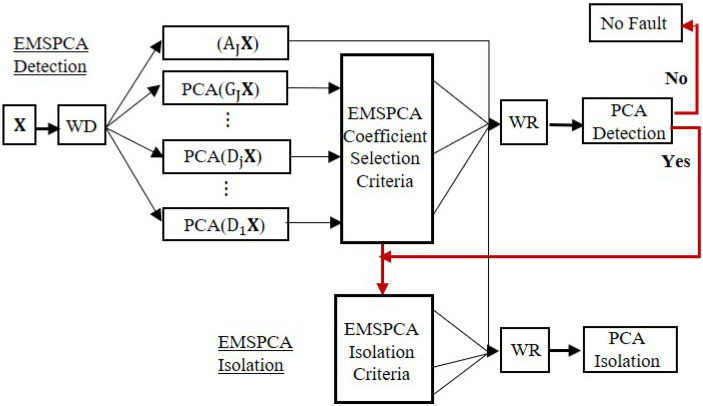
EMSPCA fault detection and isolation algorithm flow diagram.

The EMSPCA FDI algorithm involves a new coefficient selection criterion and an additional isolation block as illustrated in Figure 5.

As outlined in Figure 5, a data matrix X is decomposed with wavelet analysis (WD) into J detail scales and the J’th approximate scale. The algorithm performs PCA on all the detail scales and selects the coefficients for wavelet reconstruction by the new coefficient selection criteria. The conventional approach and the enhanced approach are described below:

- EMSPCA Coefficient Selection Criteria: Always select all coefficients of the approximate scale and select only the detail coefficients that violate the detection thresholds. Apply the same in both training and testing phases.
- MSPCA Coefficient Selection Criteria: Select all coefficients of a scale if a single limit violation occurs in that scale from the decomposed training data, and keep only the violating coefficients from the decomposed testing data. Apply the same for both details and approximate scales.

The reconstructed features from the training and testing algorithms vary considerably depending on the coefficient selection approach used. With respect to the testing data, the MSPCA method filters out the residuals in all scales, including the approximate scale, for easiest detection of a deterministic change. This approach can significantly reduce the false alarms; however, important data features in the reconstructed signal are potentially left out. One such data feature is the the approximate scale, which represents the slow-changing features of a signal and preserves the main correlation among variables. Therefore, the EMSPCA method always retains the approximate scale but filters out the residuals of the detail scales. This approach attempts to preserve the main trends of the data while reducing unnecessary stochastic features. This is similar to the approach demonstrated in [5], which does not model the approximate signal in its algorithm. Upon examining the detection performance of both techniques, results show that retaining the approximate signal is better for detecting faults. Likewise, the authors in [18] also suggest that approximate signals are effective in detecting sensor type faults.

With respect to the training data, the key difference between the coefficient selection criteria in the MSPCA and EMSPCA methods lies in how the detail scales are treated. EMSPCA identifies and retains only the violating coefficients because they represent potentially significant modeling features. If no samples violate the threshold, then the entire detail scale is removed. This is similar to the approach demonstrated by [18], where noisy signals with low energies are selectively removed. However, conventional MSPCA retains the entire scale if at least one limit violation occurs, because it deems the entire scale a significant event. As a result, the MSPCA method can produce noisier training signals depending on the number of detail scales retained. More often than desired, this causes a relatively high threshold value that fails to capture the faults in the testing data, as will be demonstrated in Section 4. Since EMSPCA does not retain entire detail scales, it generally produces noise-free signals. This leads to tighter thresholds that cause higher false alarm rates. To deal with this, the proposed algorithm will incorporate a soft-thresholding method that damps the effects of peak-like features without affecting the fault.

The new wavelet-coefficient selection approach, proposed in the EMSPCA algorithm, will address the limitations in detection performance of the conventional approach demonstrated in Section 4. This work will examine both approaches with a Monte Carlo simulation using a synthetic process model. They will also be tested on two applications: a simulated CSTR reactor and a pilot plant packed bed distillation column.

## 4. Fault Detection Performance of EMSPCA

### 4.1. Process Model and Simulation Conditions

The performances of MSPCA, EMSPCA-HT (with hard-thresholding), and EMSPCA-ST (with soft-thresholding) are assessed by a Monte Carlo simulation using the linear Gaussian model below:

(12) $$\left[\begin{array}{c} x_{1}\\ x_{2}\\ \vdots \\ x_{6} \end{array}\right]=M\left[\begin{array}{c} t_{1}\\ t_{2}\\ t_{3} \end{array}\right]+noise$$

where, $x_{1},x_{2},\ldots x_{6}$ represent process variables that are weighted functions of $t_{1}$∼N(0,1), $t_{2}$∼0.8N(0,1), and $t_{3}$∼0.6N(0,1). The elements in matrix M are randomly generated from the normal distribution N(0.2,1). M changes in every realization, which helps minimize any bias in the results attributed to a specific model. Measurement noise that follows a Gaussian distribution, 0.2N(0,1), is added to each variable. Furthermore, a fault is introduced to a random variable and at a random location in each realization to remove any biases associated with the faulty variable and the position of the fault. Two data sets are generated: a training data set (fault free) and a testing data set (faulty). The fault size is defined by a constant value times sigma, where sigma is the standard deviation of the variable in the training data. Some of the important conditions that are used in the analysis are listed below:

- Theoretical limits with 99% and 98% confidence levels are used for thresholding the detail signals, and for the detection using the reconstructed data. These confidence level values are recommended by the original MSPCA work [7].
- The number of retained principal components is 3.
- At every iteration the fault location is randomized and the process model is generated randomly.
- The number of Monte Carlo realizations is 1000.

In this work, fault detection rate (DR) and false alarm rate (FAR) are the metrics used to compare the accuracy of detection of the various techniques. Since there is a trade-off between DR and FAR, it is important to report both metrics when assessing detection accuracy. The average run length, ARL1, which quantifies the average speed of detection, will not be assessed in this work, since it is only meaningful if the FAR is fixed.

### 4.2. EMSPCA Motivation

**Figure 6:**
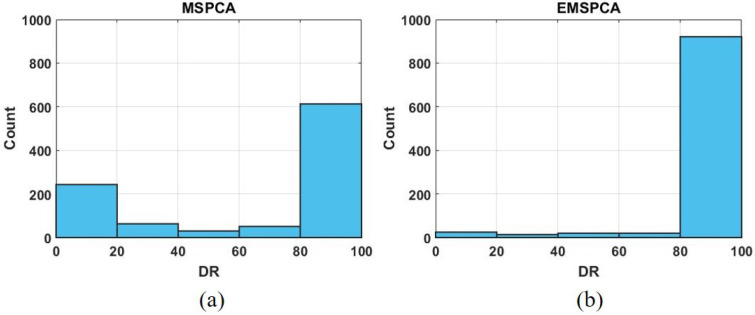
DR distributions for MSPCA (**a**) and EMSPCA (**b**); depth of 4, a fault magnitude of 1 sigma, 1000 MC runs.

This section tests MSPCA and EMSPCA algorithms with the randomized synthetic model in order to investigate the impact of the coefficient selection criteria on the detection rates. A Monte Carlo simulation consisting of 1000 runs, for a fault size of 1 sigma, and a multiscale depth of 4, was performed. A histogram of the 1000 MC runs is plotted against their detection rates in Figure 6.

**Figure 7:**
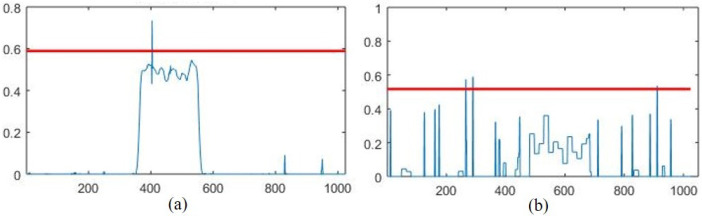
Unsuccessful detection due to: (**a**) high threshold, (**b**) poor fault projection. The red line is the detection threshold at the 95% confidence level.

As can be seen in Figure 6, the EMSPCA method significantly reduces the number of unsuccessful detections, in the <20% range, from 300 to 40 counts. The high count of unsuccessful detections is a major drawback of the MSPCA method. Poor detection performance is the outcome of two possible scenarios that are illustrated in Figure 7.

The first scenario, illustrated in Figure 7a, is characterized by a high threshold value (δ), which can cause poor detection despite a relatively large fault projection in the residual space. This is a result of retaining noisy detail scales in the training data that cause an overshoot in the prediction of the threshold. Although the fault is “detectable” due to a good-enough projection in the residual space, the detection threshold is overestimated by the MSPCA method. The second scenario, illustrated in Figure 7b, is characterized by a relatively small fault projection in the residual space ($\tilde{f}$). How well the fault is projected onto the residual space is a direct consequence of the PCA model that is built from the correlation structure of the reconstructed training data. Therefore, by always retaining the approximate signal which contains the main variations in a process signal, the model can be better preserved for the testing algorithm.

**Figure 8:**
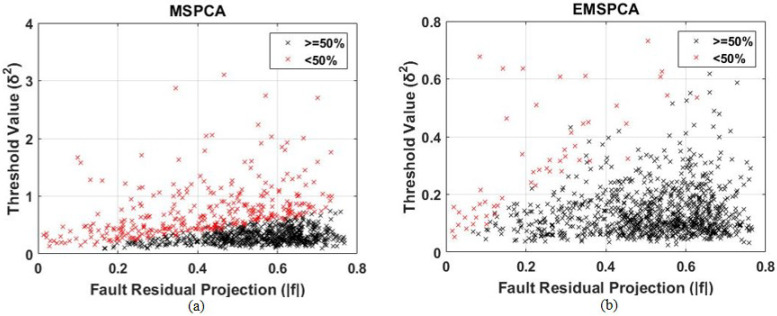
Scatter plot analysis of the relationship between threshold value and fault projection for MSPCA (**a**) and EMSPCA (**b**) for 50% success rate; depth of 4, a fault magnitude of 1 sigma, 1000 MC runs.

This section analyzes both factors, threshold value and fault projection, for all 1000 Monte Carlo runs, against their detection rates. This will help test the hypothesis that inaccurately predicted threshold values and poor fault projections are a true drawback in the coefficient selection criteria of the MSPCA method. The results are presented in a color-coded scatter plot in Figure 8. Each cross “x” indicates the result of one run, characterized by its fault projection $\tilde{f}$ (x axis) and threshold value δ (y axis). The red crosses symbolize a DR less than 50%, and the black crosses symbolize a DR greater than or equal to 50%.

**Figure 9:**
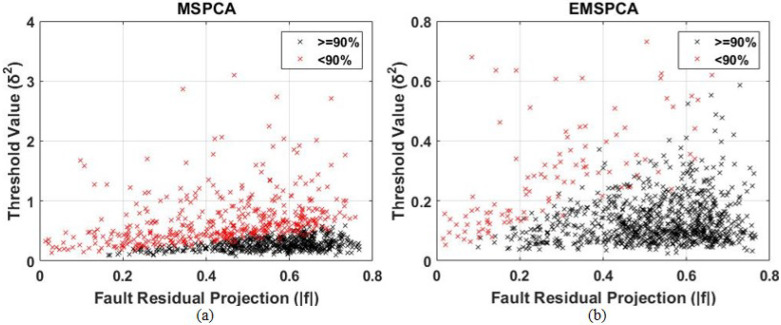
Scatter plot analysis of the relationship between threshold value and fault projection for MSPCA (**a**) and EMSPCA (**b**) for 90% success rate; depth of 4, a fault magnitude of 1 sigma, 1000 MC runs.

As can be seen in Figure 8b, the EMSPCA has a larger black area representing more successful detections when compared with that of MSPCA in Figure 8a. Therefore, EMSPCA can achieve more suitable threshold values for a wider range of fault residual projections. Furthermore, more points are concentrated in the region with higher fault residual projections, which indicates a better PCA model that can separate faulty from normal data. For a 90% success rate, the scatter plot was reproduced in Figure 9.

The linear separation presented in the scatter plots is in agreement with the “detectability criteria” posed by [27]. The criteria states that a fault is guaranteed detectable (i.e., the statistic will cross the limit) when the inequality $|\tilde{f}|>2\delta$ is true (where $|\tilde{f}|$ is the orthogonal fault projection and δ is the square-root of the threshold value). Although the figures do not demonstrate an exact proportionality of 2, they demonstrate a clear linear relationship.

### 4.3. Assessment of Fault Detection Performance of EMSPCA

**Figure 10:**
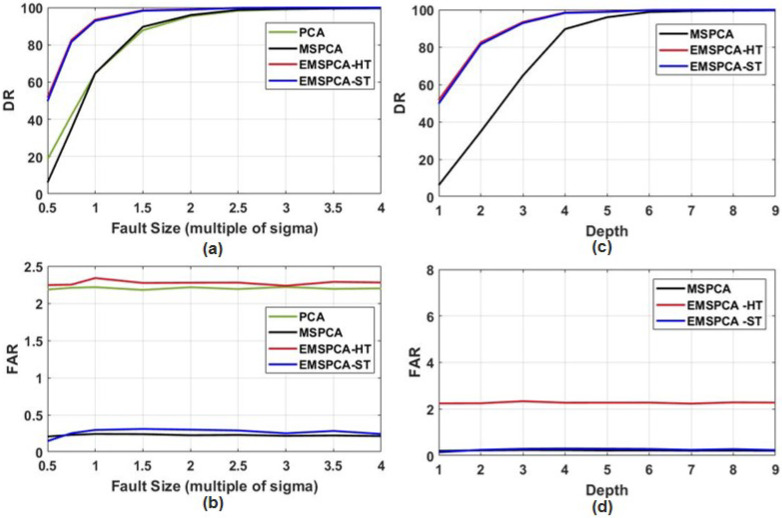
(**a**) DR and (**b**) FAR for MSPCA, EMSPCA-HT, and EMSPCA-ST across varying fault sizes and a fixed decomposition depth of 4; (**c**) DR and (**d**) FAR for MSPCA, EMSPCA-HT, and EMSPCA-ST across varying wavelet decomposition depths and a fixed unit fault size.

This section will compare the detection rate (DR) and false alarm rate (FAR) of PCA, MSPCA, EMSPCA-HT, and EMSPCA-ST for different fault sizes (represented as multiples of σ, where σ is the standard deviation of the data). The section will also study the DR and FAR for different multiscale decomposition depths. The maximum allowable depth is $log_{2}\left(N\right)$, where N is the number of samples. The choice of depth is an important parameter for MS FDI algorithms. Figure 10a,b presents the DR and FAR for different fault sizes, and Figure 10c,d presents the DR and FAR for varying decomposition depths.

As illustrated in Figure 10a,c EMSPCA can achieve significantly higher DRs for smaller fault sizes and for lower decomposition depths when compared with PCA and MSPCA. Figure 10b,d also shows that soft thresholding effectively deals with the issue of higher false alarms; EMSPCA-ST reduces the FAR of EMSPCA-HT by more than two-fold, while maintaining the same DR.Figure 10a also shows that PCA and MSPCA achieve similar DRs. However, MSPCA can achieve much better FARs, as indicated by Figure 10b. This is the benefit of the coefficeint denoising steps, that help remove all stochastic features. Although, conventional MSPCA consistently achieves very low FARs, it is at the cost of poor DR. This is apparent when the detection performance of MSPCA is compared with that of EMSPCA-ST. For brevity, “EMSPCA” will be used to refer to the technique that uses soft thresholding for the remainder of this paper.

## 5. Assessment of Fault Isolation Performance of EMSPCA

After fault detection with the EMSPCA algorithm, further coefficient denoising occurs at every scale according to the PCA isolation criteria (refer to the block diagram in Figure 5). In the conventional MSPCA algorithm, isolation is performed on the same reconstructed signal after PCA detection (no further denoising/coefficient selection). EMPCA isolation will be compared to MSPCA and PCA isolation.

**Figure 11:**
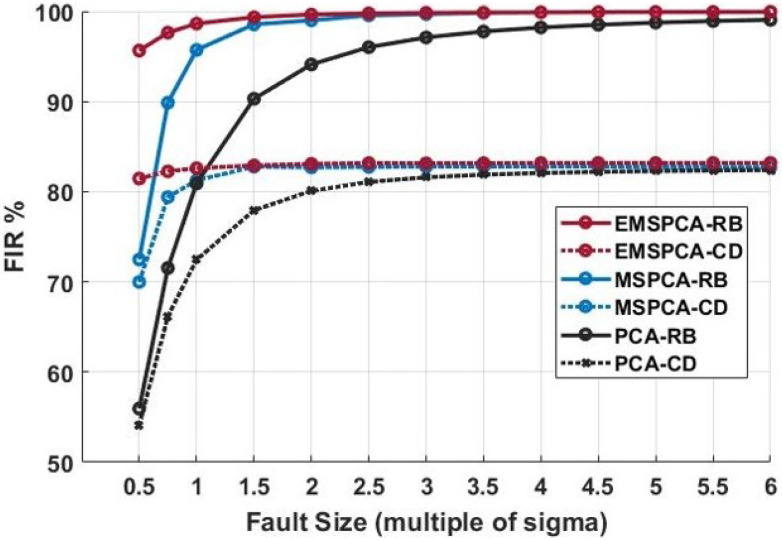
Reconstruction-based (RB) and complete decomposition (CD) fault isolation rate (FIR) for EMSPCA and PCA across varying fault sizes and a fixed multiscale decomposition depth of 4.

A novel addition of this work is to demonstrate the effectiveness of multiscale PCA reconstruction-based (RB) isolation. For comparison purposes, this section also analyzes the multiscale complete decomposition (CD) or contribution plot PCA isolation in the multiscale framework. The following simulation will obtain fault isolation rates using EMSPCA-RB, EMSPCA-CD, MSPCA-RB, MSPCA-CD, PCA-RB, and PCA-CD for varying fault sizes. The first two techniques will reveal the effect of integrating PCA isolation in a multiscale framework. For the multiscale methods, a constant decomposition depth of 4 was used, and the results are shown in Figure 11.

Figure 11 shows that the RB and CD EMPSCA methods have much higher FIRs compared to their PCA and MSPCA counterparts. This advantage is most notable for small faults where the relative contribution of noise is higher. For example, for a small fault of 0.5 sigma, EMSPCA-RB can correctly isolate it 96% of the time, MSPCA-RB can correctly isolate it 72% of the time, and PCA-RB can correctly isolate it 55% of the time. A similar trend is noted for the contribution plot or CD isolation performance. This result highlights the advantage of multiscale analysis for PCA isolation. Both MSPCA and EMSPCA improve the FIR by successfully reducing the amount of smearing and misdiagnosis. Furthermore, by integrating an isolation denoising criteria at every scale, the EMSPCA algorithm can eliminate even more fault smearing by eliminating the variable coefficients that otherwise would have caused smearing when reconstructed.

Additionally, Figure 11 demonstrates EMSPCA-RB as a much more reliable approach than EMSPCA-CD for fault isolation. For the same 0.5 sigma fault, EMSPCA-CD can isolate it 82% of the time compared with 96% for EMSPCA-RB. Moreover, the results presented are in agreement with the work of [32], which states that for a large enough fault, the RB method can guarantee correct isolation while CD cannot. As expected, the RB method is less impacted by smearing as reported in several previous works [20,37]. Furthermore, the result demonstrates the effectiveness of multiscale PCA reconstruction-based (RB) isolation using the EMSPCA algorithm.

## 6. Impact of Decimated and Undecimated Wavelet Transforms

This section implements MSPCA and EMSPCA with both wavelet transform methods; the decimated wavelet transform (DWT), which involves down-sampling, and the undecimated wavelet transform (UWT), which does not. The results were generated with a fixed fault size of 1 sigma and a Monto Carlo simulation of 1000 runs.

**Figure 12:**
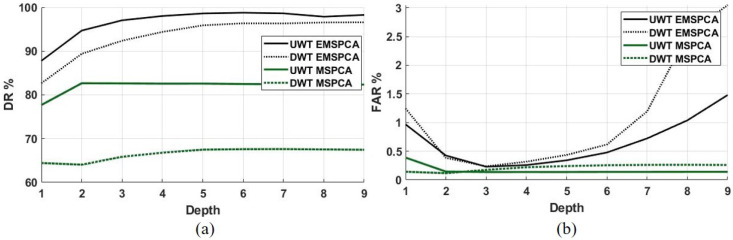
DR (**a**) and FAR (**b**) for decimated (DWT) and undecimated (UWT) EMSPCA with a fault size of 1 sigma.

**Figure 13:**
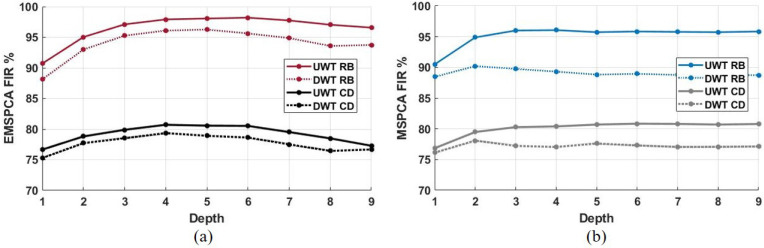
Decimated (DWT) and undecimated (UWT) comparison of RB and CD FIR across all depths and a fixed fault size of 1 sigma; (**a**) results with EMSPCA, (**b**) results with MSPCA.

Figure 12a,b shows that the detection rate and false alarm rate are improved by using the undecimated wavelet transform. The DR of EMSPCA was improved by a margin of about 5% across all decomposition depths while maintaining the same or better FAR rates when using UWT. Similarly, the UWT also improves the MSPCA DR by a margin of about 15%. The undecimated wavelet transform takes advantage of data redundancy, which provides more data and therefore more accurate statistical inferences for PCA fault detection. Furthermore, the effect of decimation, i.e., utilizing the DWT or UWT in each isolation scheme, is examined in Figure 13.

Figure 13a,b demonstrate that using the UWT instead of the DWT improves both EMSPCA and MSPCA fault isolation performance. The margin of improvement between UWT and DWT in EMSPCA is about 2–3%, while in MSPCA it is about 3–5%. The effect of UWT vs. DWT on isolation rate confirms the trade-off between data compression and modeling accuracy. The next section will further examine this trade-off by assessing the computational time of each algorithm.

## 7. Assessment of Computational Time

The trade-off between modeling accuracy and computational effort is important because the accuracy tolerance and/or the computational power available may differ from one application to another. The average computational time for fault detection and isolation is evaluated for each algorithm. Table 1 presents the average time per run next to the average fault detection, false alarm, and reconstruction-based isolation rates.

As shown by Table 1, the EMSPCA algorithm requires the most computational time compared to MSPCA and PCA. This is because EMSPCA performs reconstruction-based isolation at every scale, while MSPCA performs isolation only for the final reconstructed signal. Furthermore, the algorithms that use the decimated wavelet transform require half the time that the algorithm that uses the undecimated wavelet transform needs. However, this comes at the cost of lower monitoring performance, as indicated by the lower DR and higher FAR. Even though EMSPCA takes the most time, an average of 0.23 s per run is still considered a relatively fast algorithm.

## 8. FDI in a CSTR Reactor Using EMSPCA

**Figure 14:**
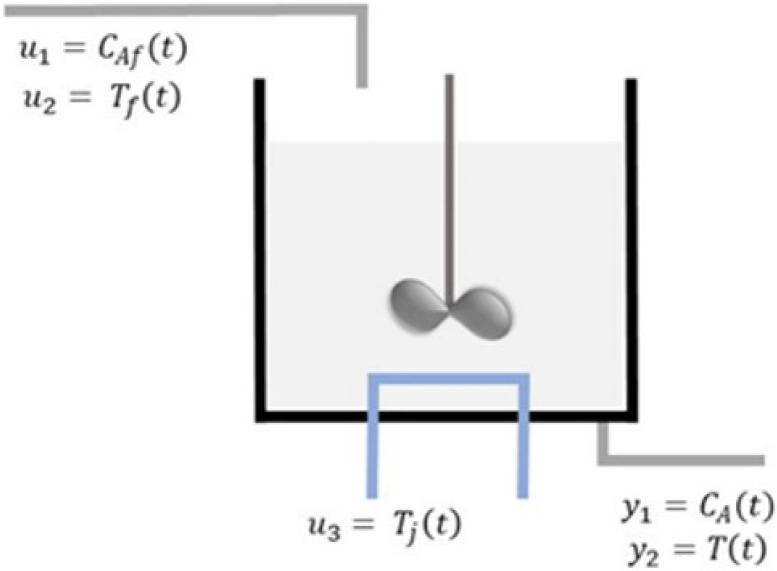
CSTR process schematic.

This section will demonstrate the proposed algorithm with a continuous stirred tank reactor process, in which an irreversible and exothermic reaction takes place. Various CSTR models have been employed in the literature, each having a unique control system (i.e., different inputs and outputs) [45,46]. The CSTR model used in this analysis was adopted from the MATLAB System Identification Toolbox [47] and described extensively in [48]. Figure 14 shows a schematic of the process.

Reactant A enters the CSTR reactor where the exothermic reaction A→B takes place. It is a steady state process; the reactor fluid is perfectly mixed, and the product leaves the reactors with uniform concentration and temperature. Since the temperature in the tank can vary significantly during the operation of the reactor, it is desirable to ensure that it remains within reasonable limits. The reactor is cooled by a surrounding jacket with coolant fluid. Concentration of A in the feed stream $C_{Af}$, temperature of the feed $T_{f}$, and the coolant jacket temperature $T_{j}$ are all inputs (manipulated variables) to the system. The inputs and outputs of the nonlinear state space model are described in (13)–(15), and the model parameters are listed in Table 2.

(13) $$u=\left[\begin{array}{c} C_{Af}\left(t\right)\\ T_{f}\left(t\right)\\ T_{j}\left(t\right) \end{array}\right];\;y=\left[\begin{array}{c} C_{A}\left(t\right)\\ T(t) \end{array}\right]$$

(14) $$\frac{dC_{A}\left(t\right)}{dt}=\frac{F}{V}\left(C_{Af}\left(t\right)-C_{A}\left(t\right)\right)-k_{0}\;exp\left(-\frac{E}{RT(t)}\right)C_{A}\left(t\right),$$

(15) $$\frac{dT(t)}{dt}=\frac{F}{V}\left(T_{f}\left(t\right)-T\left(t\right)\right)-\frac{\Delta H}{c_{p}\rho }k_{0}\;exp\left(-\frac{E}{RT(t)}\right)C_{A}\left(t\right)-\frac{UA}{c_{pj}\rho_{j}}\left(T\left(t\right)-T_{j}\left(t\right)\right).$$

**Figure 15:**
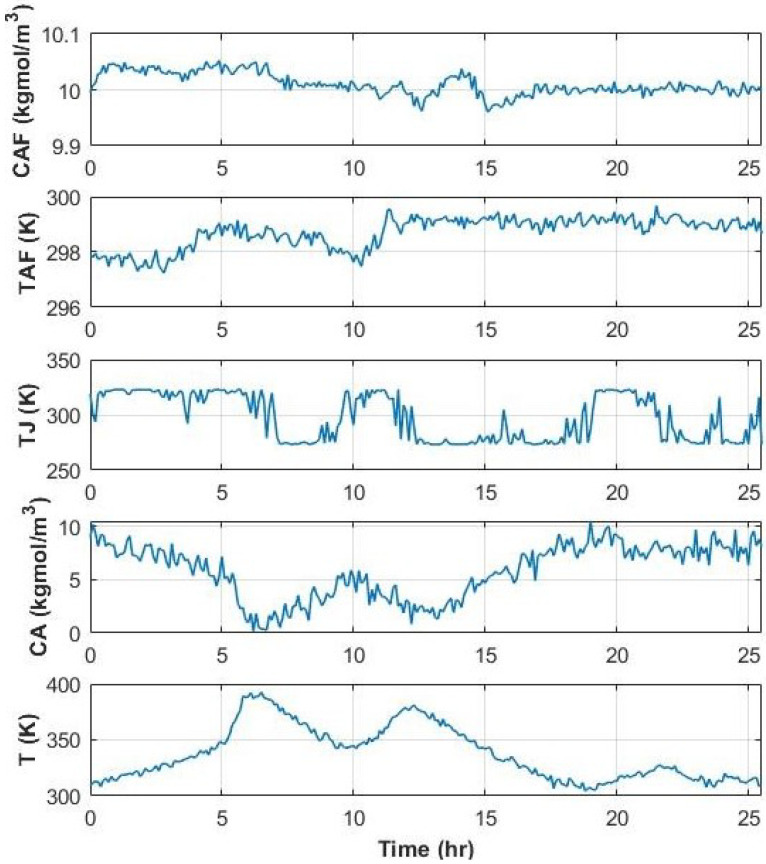
Training data inputs and outputs for the CSTR process.

The reaction and heat parameters ($k_{0},c_{p}$, and UA) have been optimized to fit the experiment [47]. The input data were generated through a MATLAB^®^ input data generation tool [47], and a nonlinear state space model was used to compute the output data. In the absence of sensor faults, the training data are illustrated in Figure 15.

**Figure 16:**
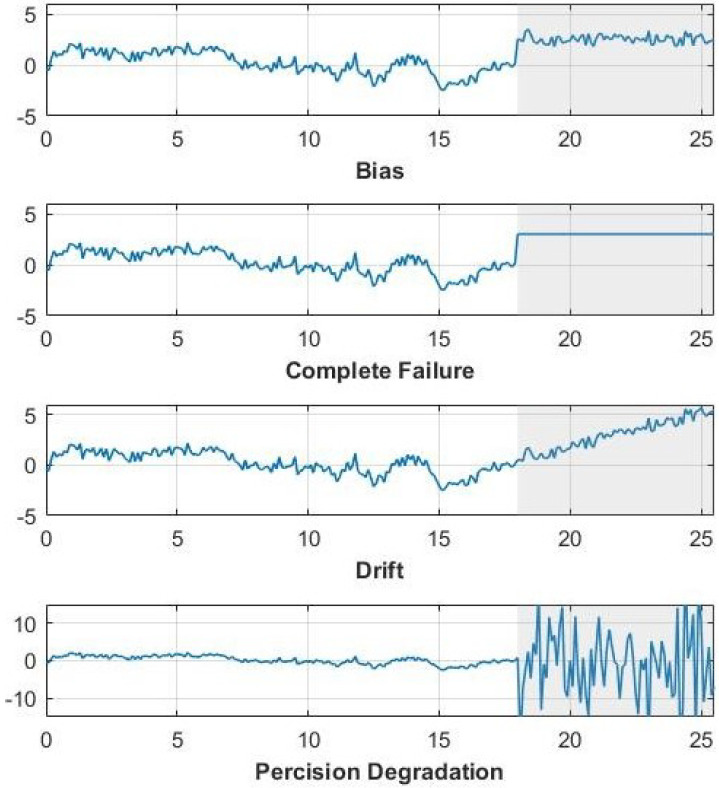
Illustration of different sensor faults in $C_{AF}$.

Process data can contain four types of sensor faults: shift in the mean (or bias), complete failure, drifting, and precision degradation. The four types of sensor faults are illustrated in Figure 16 for variable 1 of the testing data. They are generated by (16)–(19).

(16) $$F_{Bias}=X_{i}+a,$$

(17) $$F_{Drift}=X_{i}+b*t+c,$$

(18) $$F_{CompFailure}=d,$$

(19) $$F_{PerDegrad}=X_{i}+N\left(0,\sigma \right),$$

where $X_{i}$ are the samples of variable i spanning the fault location, and a,b,c,d, and σ represent constant values. $F_{Drift}$ is a fault that changes linearly with respect to variable t. For this simulation, the fault spanned 75 samples with a=3,b=0.067,c=1,d=3, and σ = 4.

In this simulation, the sensor faults are added to all variables randomly and at random locations. A Monte Carlo (MC) Simulation of 3000 runs was conducted, and the average DR and FAR are shown in Table 3.

As shown in Table 3, EMSPCA has superior DR and FAR for all types of sensor faults. Furthermore, using UWT with MSPCA significantly improves detection performance when compared to using DWT with MSPCA. However, in the EMSPCA algorithm, the improvements brought forth by using UWT over the DWT are relatively minor. Furthermore, this example demonstrates how MS representation can also be used to improve the detection performances for data sets that are highly nonlinear. The average RB and CD FIR results are shown in Table 4.

As demonstrated in Table 4, EMSPCA has superior isolation rates for both CD and RB isolation approaches compared to those of MSPCA and PCA. The EMSPCA coefficient selection criteria can effectively deal with nonlinear data and tackle the smearing problem for PCA isolation. Furthermore, the UWT is highly recommended for the MSPCA algorithm because significant isolation improvements are observed. In this example, the detection and isolation performance are significantly improved using the EMSPCA algorithm.

## 9. FDI in a Pilot Plant

**Figure 17:**
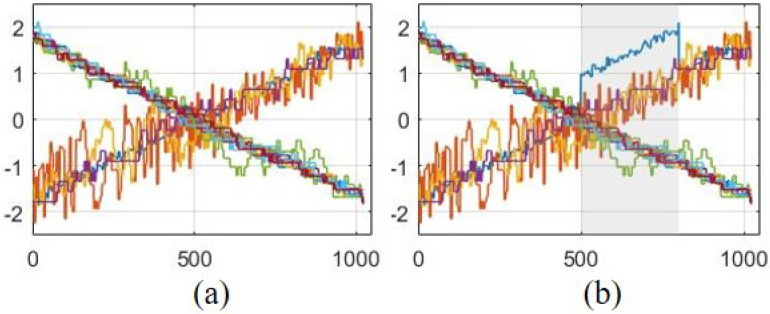
Packed bed pilot plant training (**a**) and testing (**b**) data.

In this section, the performance of the proposed EMSPCA FDI algorithm will be illustrated through its application using real experimental data from a packed-bed (PB) pilot plant operated by the Chemical Engineering Lab at Texas A&M University at Qatar. The packed column is 6 inches in diameter and 20 feet tall, with a Koch–Sulzer structured packing. A total of fourteen temperature sensors are embedded in the experimental setup. Sensor fault scenarios were replicated by adding a shift in the mean fault of varying sizes to a specific variable. For every run, the fault was embedded in a different variable and in a different location, and the average DR and FIR are computed. The normalized training data and a faulty run are illustrated in Figure 17.

**Figure 18:**
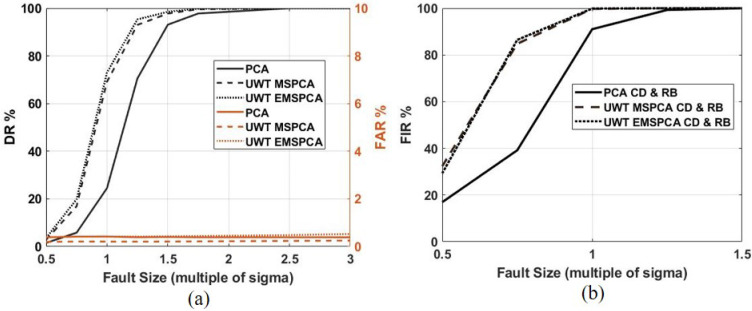
PCA, MSPCA, and EMSPCA detection and isolation performance for the pilot plant data across varying fault sizes and a fixed multiscale decomposition depth of 4; (**a**) detection rate (DR) and false alarm rate (FIR), (**b**) reconstruction-based fault isolation rate (RB FIR) and complete decomposition fault isolation rate (CD FIR).

As can be seen from Figure 17a,b, the data are highly correlated and change linearly. For this study, a CPV of 95% was used to determine the number of PCs and a 98% confidence limit for the detection threshold. For EMSPCA and MSPCA, a depth of 4 was used. The FDI results for PCA, MSPCA, and EMSPCA are plotted in Figure 18.

The results in Figure 18a,b show that EMSPCA and MSPCA have very similar DR, FAR, and FIRs. The RB and CD isolation approaches perform equally, i.e., $RB_{i}^{Q}=CD_{i}^{Q}$ for all variables. This is because the data are highly correlated and only one principle component (PC) is needed to capture 95% of the variation in the data. However, both EMSPCA and MSPCA achieve significantly higher detection and isolation rates compared to PCA. The advantages of the EMSPCA coefficient selection criteria are less notable in this specific example because of the simplicity of the data (i.e., inherently one dimensional). The more complex the system (i.e., more dimensions, more non-linear, more non gaussian), the more EMSPCA can make a difference in the FDI performance, as shown in previous examples.

## 10. Conclusions

This study provided a better understanding of the limitations of the conventional MSPCA algorithm by studying the coefficient selection criteria and its impact on detection accuracy. The proposed modifications to the coefficient selection criteria resulted in the EMSPCA algorithm, which can effectively project faults in the residual space and estimate more accurate detection thresholds. Secondly, this work extends the EMSPCA algorithm to include isolation at multiple scales with a PCA-reconstruction isolation approach. Results show large reductions in smearing that significantly improve isolation rates compared to those of MSPCA and PCA. Finally, the decimated and undecimated wavelet transforms demonstrated the trade-off between data redundancy and modeling accuracy. Furthermore, the effectiveness of the EMSPCA FDI method was demonstrated with two applications; the CSTR and the pilot plant data.

Researchers in this field are welcome to further examine the algorithm with different applications and compare with it with different techniques. Future work can develop the algorithm for a dynamic online implementation, or extend the algorithm to fault diagnosis, or develop it further for nonlinear systems with varying noise levels.

## Figures and Tables

**Table 1:** A Comparison of The Average Computational Time for Fault Detection and Isolation.

Method	Time (s/run)	DR (%)	FAR (%)	RB FIR (%)
PCA	0.01	65.21	2.15	0.79
MSPCA UWT	0.11	80.26	0.16	0.96
MSPCA DWT	0.06	65.20	0.23	0.89
EMSPCA UWT	0.23	97.21	0.25	0.97
EMSPCA DWT	0.12	93.23	0.31	0.96

**Table 2:** CSTR Model Parameters.

Symbol	Parameter Description	Units
*F*	Volumetric flow rate	m${}^{3}$/h
*V*	Volume in reactor	m${}^{3}$
$k_{0}$	Pre-exponential non-thermal factor	1/h
*E*	Activation energy	kcal/kgmol
*R*	Boltzmann’s gas constant	kcal/(kgmol K)
ΔH	Heat of reaction	kcal/kgmol
$c_{p}$	Heat capacity	kcal/(kg K)
ρ	Density	kg/m${}^{3}$
UA	Overall heat transfer coefficient times tank area	kcal/(K h)

**Table 3:** CSTR Sensor Fault Detection MC Simulation Results.

Method	Shift-In-Mean		Complete Failure		Drift		Precision Degradation		Average	
	**DR**	**FA**	**DR**	**FA**	**DR**	**FA**	**DR**	**FA**	**DR**	**FA**
PCA	70	1.7	64	1.8	64	1.8	76	1.8	69	1.8
MSPCA UWT	76	0.4	71	0.4	68	0.5	74	0.5	72	0.5
MSPCA DWT	76	1.7	72	1.8	68	1.7	76	2	73	1.8
EMSPCA UWT	95	0.4	89	0.5	82	0.5	81	0.5	87	0.5
EMSPCA DWT	93	0.5	86	0.5	80	0.5	83	0.6	86	0.5

**Table 4:** CSTR Sensor Fault Isolation Monte Carlo Simualtion Results (Of Those Detected).

Method	Shift-in-Mean		Complete Failure		Drift		Precision Degradation		Average	
	**RB**	**CD**	**RB**	**CD**	**RB**	**CD**	**RB**	**CD**	**RB**	**CD**
PCA	57	47	58	47	60	47	77	52	63	48
MSPCA UWT	66	48	68	50	70	48	79	52	71	50
MSPCA DWT	53	43	54	45	54	40	64	43	56	43
EMSPCA UWT	85	85	87	86	87	86	93	91	88	87
EMSPCA DWT	85	84	86	84	87	86	93	91	88	86

## Data Availability

The simulated CSTR data can be obtained from https://www.mathworks.com/help/ident/ug/non-adiabatic-continuous-stirred-tank-reactor-matlab-file-modeling-with-simulations-in-simulink.html (accessed on 15 July 2022).
